# Abnormal Microarchitecture and Reduced Stiffness at the Radius and Tibia in Postmenopausal Women With Fractures

**DOI:** 10.1002/jbmr.152

**Published:** 2010-06-18

**Authors:** Emily M Stein, X Sherry Liu, Thomas L Nickolas, Adi Cohen, Valerie Thomas, Donald J McMahon, Chiyuan Zhang, Perry T Yin, Felicia Cosman, Jeri Nieves, X Edward Guo, Elizabeth Shane

**Affiliations:** 1 Department of Medicine, Columbia University College of Physicians and Surgeons New York, NY, USA; 2 Helen Hayes Hospital, Clinical Research Center West Haverstraw, NY, USA; 3 Bone Bioengineering Laboratory, Department of Biomedical Engineering, Columbia University New York, NY, USA

**Keywords:** MICROARCHITECTURE, STIFFNESS, FRACTURE, OSTEOPOROSIS, POSTMENOPAUSAL

## Abstract

Measurement of areal bone mineral density (aBMD) by dual-energy x-ray absorptiometry (DXA) has been shown to predict fracture risk. High-resolution peripheral quantitative computed tomography (HR-pQCT) yields additional information about volumetric BMD (vBMD), microarchitecture, and strength that may increase understanding of fracture susceptibility. Women with (*n* = 68) and without (*n* = 101) a history of postmenopausal fragility fracture had aBMD measured by DXA and trabecular and cortical vBMD and trabecular microarchitecture of the radius and tibia measured by HR-pQCT. Finite-element analysis (FEA) of HR-pQCT scans was performed to estimate bone stiffness. DXA *T*-scores were similar in women with and without fracture at the spine, hip, and one-third radius but lower in patients with fracture at the ultradistal radius (*p* < .01). At the radius fracture, patients had lower total density, cortical thickness, trabecular density, number, thickness, higher trabecular separation and network heterogeneity (*p* < .0001 to .04). At the tibia, total, cortical, and trabecular density and cortical and trabecular thickness were lower in fracture patients (*p* < .0001 to .03). The differences between groups were greater at the radius than at the tibia for inner trabecular density, number, trabecular separation, and network heterogeneity (*p* < .01 to .05). Stiffness was reduced in fracture patients, more markedly at the radius (41% to 44%) than at the tibia (15% to 20%). Women with fractures had reduced vBMD, microarchitectural deterioration, and decreased strength. These differences were more prominent at the radius than at the tibia. HR-pQCT and FEA measurements of peripheral sites are associated with fracture prevalence and may increase understanding of the role of microarchitectural deterioration in fracture susceptibility. © 2010 American Society for Bone and Mineral Research.

## Introduction

The prevalence of osteoporosis is increasing as the population ages; it has been estimated that 10.5 million women and 3.3 million men in the United States will be affected by osteoporosis by the year 2020(1) and that the prevalence of hip fracture will double to 2.6 million by 2025.(2) These figures are of great concern because fractures are associated with significant morbidity, mortality, and health care costs.(3–5) Measurement of areal bone mineral density (aBMD) by dual-energy X-ray absorptiometry (DXA) is a powerful clinical tool and the “gold standard” for identifying those who are at increased risk of incident fracture.(6) However, since half of all postmenopausal fractures occur in women with BMD values above the World Health Organization (WHO) threshold for osteoporosis,(7,8) there is interest in investigating other methods to assess the microarchitectural determinants of bone strength and refine the prediction of fracture.

Osteoporotic fracture risk is determined both by bone strength and by risk of falling.(9) While bone strength is governed in large part by the amount of bone present, which can be assessed by measuring aBMD, many other structural and material properties contribute. Of these, microarchitecture is a major determinant of the mechanical competence or stiffness of bone(9); both trabecular (cancellous) and cortical components of microarchitecture contribute to bone strength.

High-resolution peripheral quantitative computed tomography (HR-pQCT; Xtreme CT, Scanco Medical, Bassersdorf, Switzerland) is a new, noninvasive, 3D high-resolution imaging technique that provides a true volumetric measurement of BMD (vBMD) of the distal radius and tibia. The high resolution of this technique, with its isotropic voxel size of approximately 82 µm, increases its sensitivity to microarchitectural changes that are associated with increased bone fragility.(10–12) HR-pQCT can distinguish between cortical and cancellous bone and visualize fine details of trabecular microarchitecture previously measurable only on invasive iliac crest bone biopsies. Moreover, CT data sets from individual scans can be modeled computationally by microstructural finite-element analysis (µFEA) to assess bone mechanical competence (stiffness). Several studies have demonstrated the utility of this novel technique(13–22) in elucidating microarchitectural differences between subjects with and without a history of fracture.(13,18,19,21) In addition, HR-pQCT detected substantial trabecular bone loss over time that was not detected by DXA.(23) In postmenopausal women with fractures, HR-pQCT revealed cortical thinning and decreased cancellous bone volume, with fewer, more widely spaced trabeculae and increased heterogeneity of the trabecular network.(13,19,21) µFEA of HR-pQCT scans has been shown to distinguish between postmenopausal subjects with and without wrist fractures(14,18) and to define the microarchitectural features of premenopausal women with idiopathic osteoporosis.(16,17) In this study we compared measures of BMD, microarchitecture, and trabecular bone mechanical competence (strength) in postmenopausal women with and without fragility fractures at central and peripheral sites. Specifically, we evaluated aBMD by DXA (at central and peripheral sites) and volumetric BMD (vBMD) and microarchitecture of the distal radius and tibia by HR-pQCT and FEA of the HR-pQCT data sets. We hypothesized that HR-pQCT and FEA would reveal differences in bone mass, microarchitecture, and mechanical competence between women with and without fracture.

## Methods

### Patients

Postmenopausal women over age 60 or more than 10 years postmenopause were recruited at Columbia University Medical Center (CUMC, New York, NY, USA) or the Helen Hayes Hospital (HHH, West Haverstraw, NY, USA) by advertisement or self- or physician referral. Subjects were eligible for inclusion as fracture cases if they had a documented history of a low-trauma vertebral or nonvertebral fracture that occurred after menopause. *Low trauma* was defined as equivalent to a fall from a standing height or less. Nonvertebral fractures were confirmed by review of radiographs, when possible, or radiographic reports. Vertebral fractures were identified by spine X-rays according to the semiquantitative method of Genant and colleagues.(24) Vertebrae were graded as normal or with mild, moderate, or severe deformities, defined as reductions in anterior, middle, or posterior height of 20% to 25%, 25% to 40%, and greater than 40 percent, respectively. Control subjects had no history of low-trauma fractures and no vertebral deformity on lateral radiographs. There were no BMD requirements for inclusion. Potential cases and controls were excluded if they had endocrinopathies (eg, untreated hyperthyroidism, Cushing syndrome, or prolactinoma), celiac or other gastrointestinal diseases, abnormal mineral metabolism (eg, osteomalacia, primary hyperparathyroidism), malignancy except for skin cancer, and drug exposures that could affect bone metabolism (eg, glucocorticoids, anticonvulsants, anticoagulants, methotrexate, aromatase inhibitors, or thiazolidinediones). Women using hormone-replacement therapy or raloxifene were permitted to participate. Women who had ever used teriparatide or who had taken bisphosphonates for more than 1 year were excluded. All subjects provided written informed consent, and the Institutional Review Board of Columbia University Medical Center approved this study.

Of 238 women screened, 169 were eligible and agreed to participate. The most common reasons for exclusion were bisphosphonate use for greater than 1 year (18%), subject preference not to participate (7%), age less than 60 years (5%), glucocorticoid use (3%), primary hyperparathyroidism (2%), bilateral wrist fractures, or inability to be positioned properly in the HR-pQCT scanner (2%).

### Areal bone mineral density (aBMD)

Areal BMD was measured by DXA (QDR-4500, Hologic, Inc., Walton, MA, USA, at CUMC and Lunar Prodigy, GE, Peewaukee, WI, USA, at HHH) of the lumbar spine (LS), total hip (TH), femoral neck (FN), one-third radius (1/3R), and ultradistal radius (UDR). *T*-scores compared subjects and controls with young-normal populations of the same race and sex, as provided by the manufacturer.

### HR-pQCT of the distal radius and tibia

HR-pQCT (XtremeCT, Scanco Medical AG, Bassersdorf, Switzerland) was performed by immobilizing the nondominant forearm (or nonfractured forearm in the case of prior forearm fracture) and distal tibia in a carbon fiber shell and scanning as described previously.(13,15,19) The region of interest was defined on a scout film by manual placement of a reference line at the endplate of the radius or tibia, with the first slice 9.5 and 22.5 mm proximal to the reference line at the radius and tibia, respectively. A stack of 110 parallel CT slices was acquired at the distal end of both sites using an effective energy of 40 keV, image matrix size of 1024 × 1024, with a nominal voxel size of 82 µm. This provided a 3D image of approximately 9 mm in the axial direction. Attenuation data were converted to equivalent hydroxyapatite (HA) densities. The European Forearm Phantom was scanned regularly for quality control.

The analysis methods have been described, validated,(25–27) and applied in several recent clinical studies.(13,14,16–23,28,29) Briefly, the volume of interest (VOI) was automatically separated into cortical and trabecular regions using a threshold-based algorithm set to one-third the apparent cortical bone density (*D*_cort_). Mean cortical thickness (Ct.Th) was defined as the mean cortical volume divided by the outer bone surface. Trabecular bone density (*D*_trab_) was defined as the average bone density within the trabecular VOI and BV/TV (%) derived from *D*_trab_, assuming that the density of fully mineralized bone is 1.2 g hydroxyapatite/cm^3^ (BV/TV^*d*^ = 100 × *D*_trab_/1200 mg HA/cm^3^). Inner trabecular density (*D*_inn_) was defined as the inner 60%, and the meta trabecular density (*D*_meta_) was defined as the outer 40% of the trabecular region. Since measurements of trabecular microstructure are limited by the resolution of the XtremeCT, which approximates the width of individual trabeculae, trabecular structure was assessed using a semiderived algorithm.(10,25) Trabeculae were identified by a medial-axis transformation method, and the distance between them was assessed by the distance-transform method.(11,30) Tb.N* was defined as the inverse of the mean spacing of the medial axes. Tb.Th and Tb.Sp then were derived from BV/TV^d^ and Tb.N* using formulas from traditional quantitative histomorphometry; Tb.Th = (BV/TV^*d*^)/Tb.N* and Tb.Sp = (1 – BV/TV^*d*^)/Tb.N*.

### HR-pQCT image-based µFEA

HR-pQCT data was used to calculate apparent anisotropic elastic moduli of trabecular bone, a surrogate measure of bone's resistance to force, otherwise termed *mechanical competence* or *stiffness*, as we have described previously.(15–17,26) First, the mineralized phase was thresholded automatically by using a Laplace-Hamming filter followed by global threshold using a fixed value of 40% of maximal grayscale value of the images. Then a VOI of 70 × 70 × 70 voxels, corresponding to 5.74 × 5.74 × 5.74 mm^3^, was isolated manually from the center of each thresholded radius image, and a VOI of 110 × 110 × 110 voxels corresponding to 9.02 × 9.02 × 9.02 mm^3^ was isolated manually from the center of each thresholded tibial image. The location of the VOI was defined by the center of the largest cylinder that could fit within the trabecular compartment, providing a reproducible location based on a customized protocol. Each subvolume of HR-pQCT image of the distal radius and distal tibia was converted to a µFE model by directly converting bone voxels to 8-node elastic brick elements with an element size of 82 × 82 × 82 µm^3^. Bone tissue properties were assumed to be isotropic and linearly elastic with a Young's modulus of 15 GPa and a Poisson's ratio of 0.3 for all models.(31) Six µFE analyses representing three uniaxial compressions and three shear compressions were performed on each model using an element-by-element precondition conjugate gradient solver.(32) Based on the anisotropic compliance matrix, estimated apparent elastic constants (three apparent Young's moduli, *E*_11_, *E*_22_, and *E*_33_) were calculated and sorted.(33,34) *E*_11_ represents the modulus along the mediolateral direction, *E*_22_ along the anterior-posterior direction, and E_33_ along the axial direction. The anisotropic compliance matrix also was used to calculate three shear moduli (*G*_23_, *G*_31_, and *G*_12_).

### Statistical methods

Analyses were conducted with STATA Version 9.0 (Stata Corp., College Station, TX, usa) and SAS Version 9.1 (SAS Institute, Inc., Cary, NC, USA). Two-sided *p* values of less than .05 were considered to indicate statistical significance. Descriptive data are presented as mean ± SD and group comparisons as mean ± standard error of the mean (SEM). Differences between fracture and nonfracture subjects were assessed by Student's *t* test or the chi-square test. ANOVA was used to evaluate differences in HR-pQCT parameters at the radius or tibia after adjustment for aBMD *T*-score at the ultradistal radius or total hip, respectively. Regression analyses were performed to investigate the effects of race. Standard receiver operator characteristic (ROC) curve analysis was performed to determine the ability of DXA and HR-pQCT to discriminate fracture status. In this type of analysis an area under the curve (*AUC*) of more than 0.75 is considered compelling evidence for the ability to discriminate an outcome. A diagnostic test with an *AUC* of 0.5 is considered to perform no better than chance.

## Results

### Subject characteristics

Of 169 women enrolled (mean age 68 ± 7 years), 68 had a history of postmenopausal fragility fracture (Table 1). Subjects were racially diverse: 78% white, 16% Hispanic, 4% African American, and 2% from other backgrounds. The most common sites of fracture were forearm (25, 37%), spine (20, 29%), ankle (13, 19%), metatarsal (11, 16%), and humerus (4, 6%). There also were 3 subjects with hip fractures and 3 with rib fractures. Seventeen subjects (25%) had sustained multiple postmenopausal fractures. Subjects with and without fractures did not differ on the basis of age, body mass index (BMI), or ethnicity. Mean age at menopause (49 ± 5 years) and time since menopause (∼19 years) did not differ between subjects with and without fractures. The average time between symptomatic fracture and study evaluation was 5.5 ± 5.6 years. Women enrolled were ambulatory and generally in good health. Fracture and nonfracture subjects were well matched on demographic factors and medical conditions, including diabetes, hypertension, heart disease, and thyroid disease. Family history of osteoporosis and fractures, alcohol and tobacco use, and medication and supplement use, notably use of calcium and vitamin D supplements, hormone-replacement therapy, raloxifene, and bisphosphonates did not differ between the groups. Thyroxine use was significantly greater among nonfracture subjects.

**Table 1:** Characteristics of the Study Population (mean ± SEM)

	Fracture (*n* = 68)	Nonfracture (*n* = 101)	*p* Value
Age (years)	69 ± 1	68 ± 1	.30
Race % white	81%	76%	.93
% African American	3%	5%	
% Hispanic	15%	17%	
% Other	1%	2%	
Height (cm)	160 ± 1	161 ± 1	.36
Weight (kg)	67 ± 2	68 ± 2	.60
Years since menopause	20 ± 1	18 ± 1	.34
Oophorectomy (%)	18%	18%	.99
Family history of osteoporosis (%)	49%	44%	.63
Family history of fracture (%)	38%	36%	.87
Tobacco use,			0.85
Pack-years	22 ± 5	21 ± 3	
Never (%)	50%	44%	
Former (%)	50%	54%	
Current (%)	0%	1%	
Alcohol use (beverages per day)	1 ± 0	1 ± 0	.99
Medication use			
Calcium supplements, total daily dose (mg)	608 ± 78	612 ± 60	.97
Vitamin D supplements, total daily dose (IU)	568 ± 78	797 ± 135	.19
Hormone- replacement therapy			
Past (%)	42%	46%	0.87
Current (%)	3%	6%	67
Bisphosphonates-			
Past (%)	6%	5%	.99
Current (%)	4%	1%	.46
Raloxifene (%)	6%	2%	.37
Thyroxine (%)	8%	22%	.02

### Areal bone mineral density

**Fig. 1:**
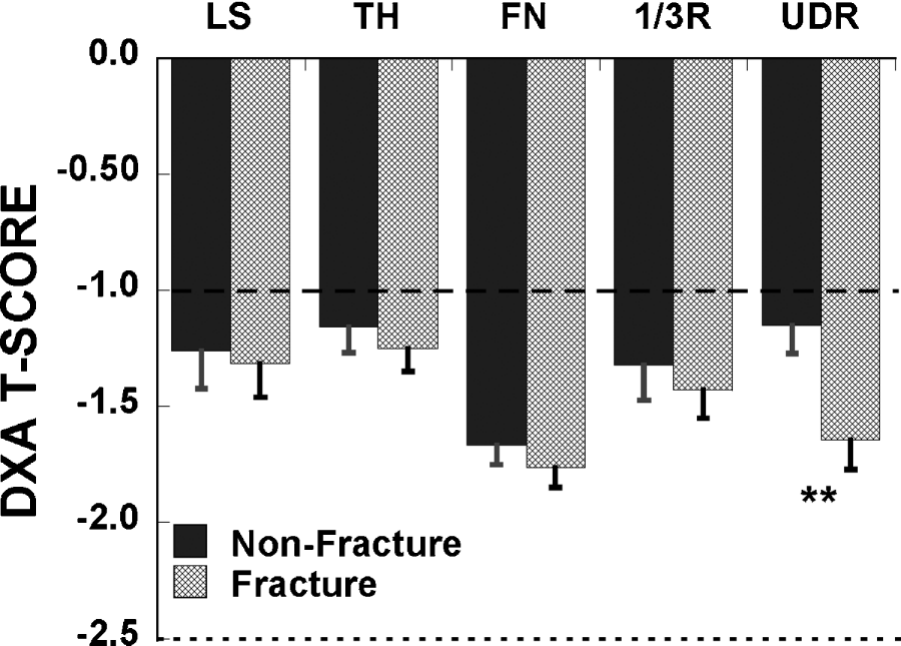
Comparison of *T*-scores by DXA in postmenopausal women with and without fragility fractures. No significant differences at any site except for the ultradistal radius (*p < .01). Horizontal lines indicate the WHO thresholds for low bone mass (*dashed line*) and osteoporosis (*dotted line*).

Mean aBMD by DXA was well above the WHO osteoporosis threshold (*T*-score ≤ −2.5) in the vast majority of women, those both with and without fractures (Fig. 1). The prevalence of osteopenia at any site was 56% among fracture subjects and 49% among nonfracture subjects. The prevalence of osteoporosis at any site was 38% among fracture subjects and 40% among nonfracture subjects. Mean *T*-scores at the LS, TH, and 1/3R were similar in women with and without fracture. In contrast, at the ultradistal radius, the mean T-score was 0.5 SD lower in women with fractures (*p* < .01; Fig. 1). Bone mineral apparent density (BMAD; BMD/square root of bone area) was calculated at the LS, FN, and 1/3R to control for the effects of bone size. There were no differences in BMAD between fracture patients and controls (data not shown).

### vBMD and microarchitecture by HR-pQCT

**Fig. 2:**
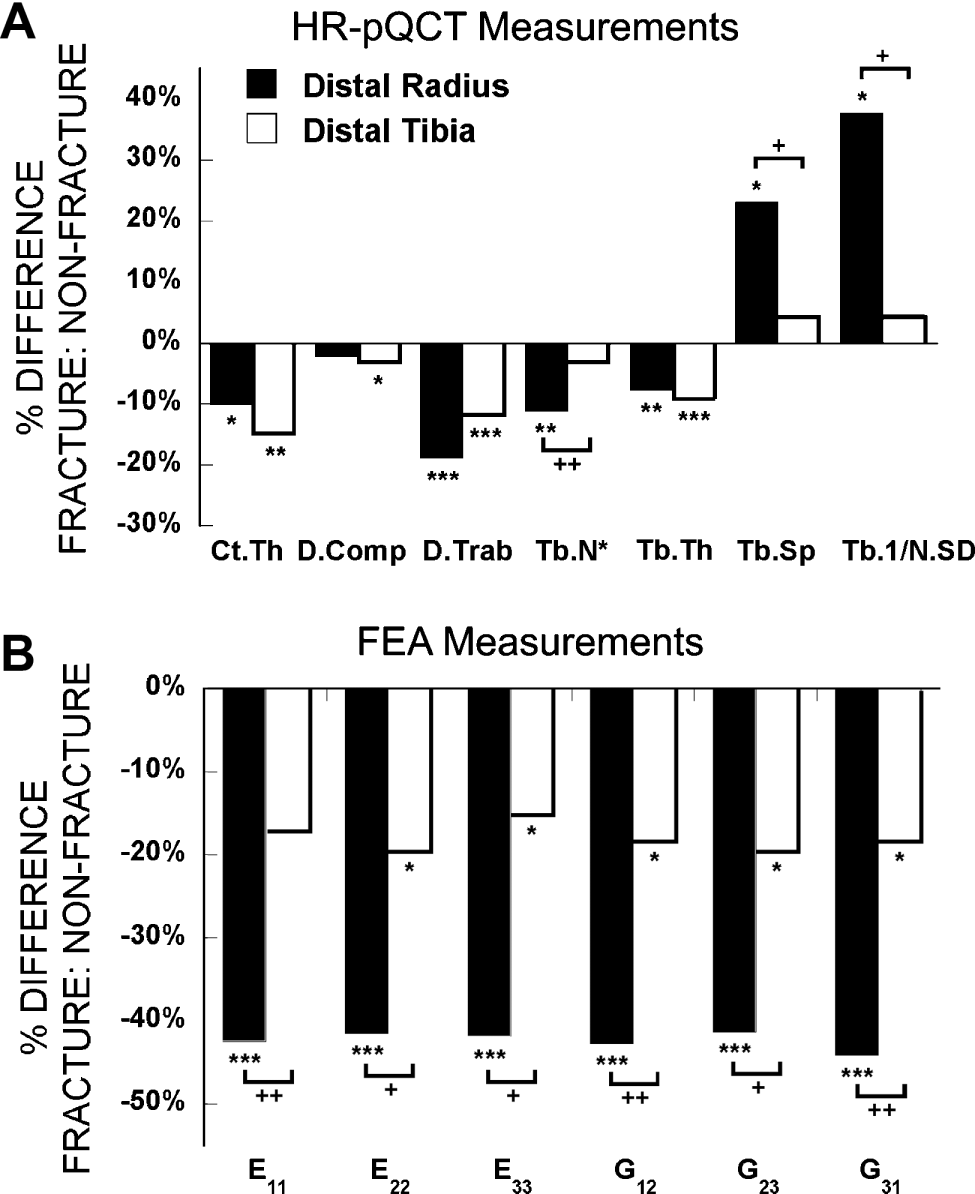
Comparison of the percent difference in HR-pQCT (*A*) and FEA (*B*) measurements between fracture and nonfracture subjects at the distal radius (*filled bars*) and tibia (*open bars*; *p < .05, **p < .01, ***p < .001 for comparisons between fracture and nonfracture subjects, and ^+^*p* < .05, ^++^*p* < .01 for comparisons between radius and tibia). Ct.Th = cortical thickness; D.comp = cortical density; D.Trab = trabecular density; Tb.N* = trabecular number; Tb.Sp = trabecular separation; Tb.1/NSD = network inhomogeneity; *E*_11_, *E*_22_, *E*_33_ = Young's moduli; *G*_12_, *G*_23_, *G*_31_ = shear moduli.

In contrast to the DXA findings, vBMD, cortical and trabecular microarchitecture differed markedly between fracture and nonfracture subjects at both the radius and the tibia (Table 2). At the radius, total density was 13% lower in fracture subjects. Fracture subjects had 10% lower cortical thickness, whereas differences in cortical density were not significant. Trabecular density and all microarchitectural parameters (ie, trabecular number, thickness, and separation and heterogeneity of the network) differed significantly by 8% to 38% in women with fractures (Fig. 2*A*).

**Table 2:** Comparison of Volumetric Density, Microstructure, and Mechanical Parameters in Subjects With and Without Fractures

	Fracture, mean ± SEM	Nonfracture, mean ± SEM	Odds ratio (OR) (95% confidence interval)	*p* Value
HR-pQCT—Radius				
Total bone density (mgHA/cm^3^)	261.7 ± 7.0	300.3 ± 7.5	0.527 (0.360, 0.771)	<0.001*
Trabecular bone density (mgHA/cm^3^)	107.0 ± 4.4	132.0 ± 4.1	0.489 (0.335, 0.715)	<0.0001**
Cortical bone density (mgHA/cm^3^)	833.3 ± 8.5	852.1 ± 7.3	0.766 (0.558, 1.051)	0.10
BV/TV (%)	8.9 ± 0.4	11.0 ± 0.3	0.490 (0.336, 0.715)	<0.0001**
Number of trabeculae (1/mm)	1.58 ± 0.05	1.78 ± 0.04	0.590 (0.420, 0.828)	<0.01*
Trabecular thickness (mm)	0.057 ± 0.001	0.061 ± 0.001	0.627 (0.446, 0.881)	<0.01
Trabecular separation (mm)	0.66 ± 0.05	0.54 ± 0.02	1.712 (1.084, 2.704)	0.02*
Inhomogeneity (mm)	0.38 ± 0.04	0.27 ± 0.03	1.451 (1.001, 2.103)	0.04
Cortical thickness (mm)	0.65 ± 0.02	0.72 ± 0.02	0.652 (0.464, 0.916)	0.01
Total area (cm^2^)	232.0 ± 5.0	223.0 ± 3.9	1.257 (0.918, 1.721)	0.15
FEA (MPa)—Radius				
*E*_11_	160.8 ± 18.3	279.4 ± 20.4	0.434 (0.278, 0.678)	<.0001**
*E*_22_	248.0 ± 29.4	424.1 ± 33.8	0.468 (0.300, 0.729)	<.001**
*E*_33_	445.5 ± 54.4	766.3 ± 58.4	0.480 (0.317, 0.727)	<.0001**
*G*_23_	129.1 ± 15.6	220.0 ± 17.6	0.486 (0.318, 0.744)	<.001**
*G*_31_	84.6 ± 10.3	151.4 ± 11.8	0.446 (0.287, 0.692)	<.0001**
*G*_12_	73.0 ± 8.4	127.4 ± 9.9	0.447 (0.286, 0.698)	<.0001**
HRpQCT—Tibia				
Total bone density (mgHA/cm^3^)	214.7 ± 5.0	244.9 ± 5.1	0.485 (0.330, 0.713)	<.0001****
Trabecular bone density (mgHA/cm^3^)	130.2 ± 3.7	147.6 ± 3.4	0.566 (0.399, 0.803)	<.001***
Cortical bone density (mgHA/cm^3^)	760.2 ± 8.6	784.3 ± 6.7	0.699 (0.506, 0.966)	.03*
BV/TV (%)	10.8 ± 0.003	12.3 ± 0.003	0.566 (0.398, 0.803)	<.001***
Number of trabeculae (1/mm)	1.70 ± 0.04	1.76 ± 0.03	0.839 (0.612, 1.149)	.27
Trabecular thickness (mm)	0.064 ± 0.001	0.071 ± 0.001	0.568 (0.404, 0.800)	<.001***
Trabecular separation (mm)	0.54 ± 0.01	0.52 ± 0.03	1.192 (0.874, 1.626)	.27
Inhomogeneity (mm)	0.26 ± 0.01	0.25 ± 0.01	1.092 (0.804, 1.484)	.57
Cortical thickness (mm)	0.74 ± 0.03	0.86 ± 0.03	0.563 (0.389, 0.814)	<.01**
Total area (cm^2^)	686.2 ± 14.6	662.8 ± 10.1	1.241 (0.908, 1.697)	.17
FEA (MPa)—Tibia				
*E*_11_	175.9 ± 12.7	212.1 ± 13.5	0.731 (0.520, 1.026)	.052
*E*_22_	257.3 ± 18.6	320.1 ± 19.8	0.690 (0.491, 0.972)	.02*
*E*_33_	766.5 ± 46.6	901.8 ± 45.9	0.722 (0.520, 1.001)	<.05
*G*_23_	141.0 ± 10.3	175.3 ± 10.7	0.691 (0.493, 0.968)	.02*
*G*_31_	102.2 ± 7.1	124.9 ± 7.8	0.710 (0.507, 0.995)	.03
*G*_12_	82.3 ± 6.1	100.9 ± 6.1	0.707 (0.505, 0.990)	.04

Note: Asterisks denote significance of comparisons after adjustment for UDR (radius) and TH (tibia) *T*-score.

* *p* < .05

** *p* < .01

*** *p* < .001, and

**** *p* < .0001.

Significant differences also were observed at the tibia, but the pattern differed from that of the radius (Table 2 and Fig. 2*A*). Total, cortical, and trabecular density all were significantly lower by 3% to 12% in fracture subjects. In terms of microarchitectural parameters, only cortical thickness and trabecular thickness were lower in fracture subjects, by 15% and 9%, respectively. Compared with radial measurements, the percent differences between fracture and nonfracture groups were significantly less pronounced at the tibia for trabecular number and separation and heterogeneity of the network (Fig. 2*A*).

### Estimated stiffness by FEA

Trabecular bone stiffness was significantly and substantially lower in women with fractures than in controls at both sites and in all directions except the mediolateral direction (*E*_11_) at the tibia (Table 2, Fig. 2*B*). Differences were more pronounced at the radius, where both Young's moduli and shear moduli were more than 40% lower in fracture subjects. These differences between groups were significantly greater than those found at the distal tibia, where Young's moduli and shear moduli were 15% to 20% lower in women with fractures.

**Fig. 3:**
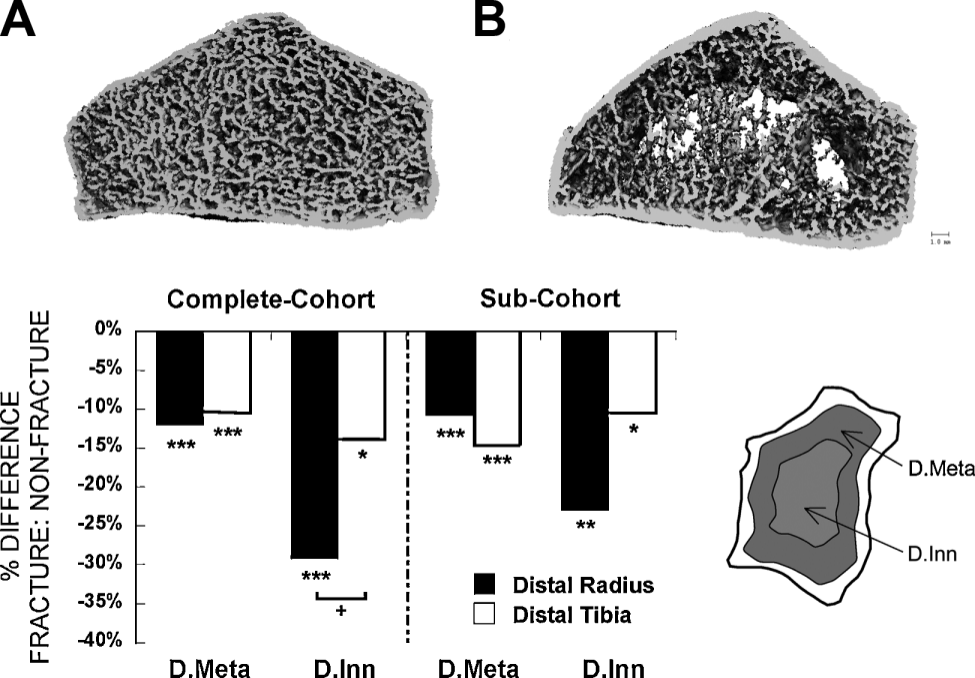
HR-pQCT scans from fracture subjects with (*A*) and without (*B*) measurable inner trabecular density and FEA at the distal radius (*filled bars*) and tibia (*open bars*). (*C*) Comparison of the percent difference in inner trabecular density (*D*_inn_) and metatrabecular density (*D*_meta_), detailed in the schematic on the right, between fracture and nonfracture subjects in the entire cohort and in a subcohort excluding those subjects without measurable inner trabecular density and FEA (*p <.05, **p < 0.01, ***p < .001 for comparisons between fracture and nonfracture subjects. ^+^*p* < .05 for comparison between radius and tibia).

Fifteen subjects had so few central trabeculae that trabecular stiffness could not be analyzed by FEA. Representative HR-pQCT scans from fracture subjects with and without measurable inner trabecular density are shown in Fig. 3*A, B*, respectively. Both inner (*D*_inn_) and outer (*D*_meta_) trabecular bone density were significantly lower in fracture than in nonfracture groups regardless of whether women without measureable *D*_inn_ were included (Fig. 3*C*, *right panel*) or excluded (Fig. 3*C*, *left panel*).

The pattern of deterioration of inner and outer trabecular bone differed between the radius and the tibia. At the radius, the loss of central (inner) trabecular bone exceeded that observed in the outer subcortical region; in contrast, at the tibia, trabecular loss was more uniform across inner and outer regions and less severe than at the radius (Fig. 3*C*). At the radius, more subjects in the fracture group (9 versus 4) had no analyzable data for FEA because of lack of inner trabeculae. In contrast, only one subject in each group lacked measureable trabecular structure at the tibia. When subjects without measureable central trabecular structure were excluded from FEA analysis of HR-pQCT results, the overall FEA results did not change significantly.

Radial HR-pQCT and FEA parameters were assessed after controlling for aBMD *T*-score at the ultradistal radius, the only site at which aBMD differed significantly between fracture and nonfracture subjects. Total density, trabecular density, bone volume fraction, number, and separation remained lower in fracture subjects (Table 2). Differences in cortical thickness and trabecular thickness and inhomogeneity were no longer significant. Differences in FEA parameters at the radius did not change after adjustment. Adjustment for aBMD *T*-score at the total hip did not alter differences observed in tibial HR-pQCT scans between fracture and nonfracture subjects. However, after adjustment for TH BMD *T*-score, only *E*_22_ and *G*_23_ remained significantly different between fracture and nonfracture subjects. Adjusting for race (white versus nonwhite) and limiting the sample to subjects not on antiresorptive treatment (ie, hormone-replacement therapy, raloxifene, or bisphosphonates) did not alter the differences between fracture and nonfracture subjects. By ROC analysis, discrimination of fracture status by DXA and HR-pQCT parameters was not statistically different (*AUC* 0.51–0.62 for DXA measures, 0.55–0.70 for HR-pQCT and FEA measures). Thus, despite the highly significant between-groups differences, no DXA, HR-pQCT, or FEA parameters demonstrated sufficient fracture discrimination between groups by ROC analysis (*AUC* > 0.75; Table 3).

**Table 3:** ROC Analysis of Fracture Prediction by DXA, HR-pQCT, and FEA

	*AUC*
DXA	
Lumbar spine (L_1_–L_4_) (g/cm^2^)	0.53
Total hip (g/cm^2^)	0.53
Femoral neck (g/cm^2^)	0.55
1/3 Radius (g/cm^2^)	0.51
Ultradistal radius (g/cm^2^)	0.62
HR-pQCT—Radius	
Total bone density (mgHA/cm^3^)	0.67
Trabecular bone density (mgHA/cm^3^)	0.67
Cortical bone density (mgHA/cm^3^)	0.59
BV/TV (%)	0.67
Number of trabeculae (1/mm)	0.63
Trabecular thickness (mm)	0.62
Trabecular separation (mm)	0.64
Inhomogeneity (mm)	0.63
Cortical thickness (mm)	0.63
Total area (cm^2^)	0.56
FEA—Radius	
*E*_11_	0.70
*E*_22_	0.69
*E*_33_	0.69
*G*_23_	0.68
*G*_31_	0.70
*G*_12_	0.69
HR-pQCT—Tibia	
Total bone density (mg HA/cm^3^)	0.66
Trabecular bone density (mg HA/cm^3^)	0.63
Cortical bone density (mg HA/cm^3^)	0.58
BV/TV (%)	0.63
Number of trabeculae (1/mm)	0.55
Trabecular thickness (mm)	0.66
Trabecular separation (mm)	0.57
Inhomogeniety (mm)	0.55
Cortical thickness (mm)	0.64
Total area (cm^2^)	0.55
FEA—Tibia	
*E*_11_	0.57
*E*_22_	0.58
*E*_33_	0.58
*G*_23_	0.58
*G*_31_	0.57
*G*_12_	0.58

## Discussion

In this study we found that postmenopausal women with a variety of central and peripheral fragility fractures had substantial and highly significant differences in vBMD, microarchitecture, and bone mechanical properties compared with those without fractures. We extend previous work by describing differences in the pattern of deterioration and decreased strength at the radius and the tibia. Women with a history of fracture had lower vBMD values and microarchitectural deterioration, with thinner cortices and fewer, thinner, more widely and unevenly spaced trabeculae. Women with fractures had markedly reduced trabecular stiffness. Although these differences were apparent at both sites and involved both cortical and trabecular compartments, they were more prominent at the radius than at the tibia. Contrasting with the profound distinctions observed by HR-pQCT and FEA, no difference was detected by DXA at any of the sites (ie, LS, TH, FN, and 1/3R) typically used for diagnosis of osteoporosis. Only at the UDR were significant differences between fracture and nonfracture subjects observed. Despite marked differences in vBMD and microarchitecture, no DXA, HR-pQCT, or FEA parameter demonstrated sensitivity or specificity for fracture discrimination. However, HR-pQCT and FEA provide insight into microarchitectural differences that may underlie susceptibility to fracture in postmenopausal women.

A number of recent studies have demonstrated the utility of HR-pQCT to detect microarchitectural deterioration in patients with fractures and to detect bone loss over time.(13,14,18–21,23,28,29) Boutroy and colleagues found that an array of radial HR-pQCT trabecular parameters differed between osteopenic postmenopausal women with and without fractures.(13) In contrast to our finding that tibial HR-pQCT measurements differ by fracture status, they did not detect differences in HR-pQCT measurements at the tibia, possibly because most of their subjects had forearm fractures or because our study was not restricted to women with osteopenia.(13) Similar to our results, other recent studies that included more patients with central fractures did detect differences in tibial HR-pQCT in subjects with fractures.(19–21)

In addition to substantial differences in radial and tibial vBMD, microarchitecture, and stiffness between subjects with and without fractures, we found that the pattern of differences varied at the radius and the tibia. vBMD, trabecular density, trabecular thickness, and cortical thickness differed between fracture and nonfracture subjects at both sites. However, at the radius, the pattern was more consistent with trabecular loss, with profound reductions in trabecular number and increased network inhomogeneity. At the tibia, cortical density was more severely affected, and although trabecular thickness was lower, trabecular number did not differ, suggesting predominantly cortical losses. This pattern is similar to that reported by Sornay-Rendu.(19) The discrepancies between radius and tibia may be due to differences in the ratio of plates to rods, which may vary by skeletal site, but also could be related to loading, because the tibia is weight-bearing and the radius is not. That fracture subjects had markedly lower inner trabecular density at the radius than the tibia suggests that loss of inner trabecular structure may be offset by weight bearing. Conversely, the predominance of cortical findings at the tibia suggests that cortical bone loss may not be offset by weight bearing. Although we had insufficient power to examine subjects with vertebral or hip fractures separately, our ability to detect differences at peripheral sites in a group of women with both central and peripheral fractures provides further evidence that HR-pQCT parameters reflect ubiquitous skeletal changes, not just deterioration at peripheral sites. In this regard, recently we have reported significant correlations between estimates of stiffness by FEA based on CT scans of central (spine and hip) and peripheral (radius and tibia) sites.(35)

Somewhat surprisingly, fracture and nonfracture subjects were similar with regard to common risk factors for osteoporosis, including BMI and family history of fracture. This similarity may reflect selection bias because women with common risk factors for osteoporosis may be more interested in participating in a study of this type. Similarly, we did not observe a difference in BMD measured by DXA between fracture and nonfracture subjects at any site except the ultradistal radius. Our results suggest that HR-pQCT detects differences in vBMD and structural mechanisms that underlie the pathophysiology of fracture in postmenopausal women and that are not measured by DXA. Some HR-pQCT studies found that aBMD was the same in fracture and nonfracture groups,(13) whereasothers have found lower aBMD measurements.(19,21) The few studies that have reported UDR BMD found that it was lower in fracture subjects.(14,18,19,29) Our finding raises the question of whether UDR BMD may be a useful and clinically relevant site for fracture discrimination. However, whereas Melton and colleagues found that UDR aBMD discriminated cases from controls better than 1/3R, FN aBMD was the most significant predictor of Colles' fracture risk in a multivariate analysis.(29) We also observed that radial HR-pQCT measurements were influenced by BMD. After adjusting for aBMD *T*-score at the ultradistal radius, differences in some trabecular microstructure parameters and cortical thickness were attenuated, whereas overall density, trabecular density, bone volume fraction, and trabecular number remained significantly lower and trabecular separation remained significantly higher in fracture subjects. FEA parameters did not change. At the tibia, adjustment for total-hip *T*-score did not affect group differences in HR-pQCT at all but attenuated FEA differences. Our results, similar to reports by others,(19) suggest that aBMD measured by DXA and vBMD and microarchitecture measured by HR-pQCT are independently associated with fracture.

Trabecular stiffness by FEA, a surrogate measure of bone strength, has excellent agreement with true biomechanical tests.(37–39) Two recent FEA studies have demonstrated that postmenopausal women with a history of radius fragility fractures(14,18) have abnormal microarchitecture, reduced stiffness, and increased failure load compared with nonfracture subjects. Premenopausal women with idiopathic osteoporosis, manifested as low BMD or fragility fractures, had reduced stiffness at the radius and tibia compared with premenopausal controls.(16) In a recent large study of wrist fracture patients, Melton and colleagues reported that the bone-strength variable that best predicted forearm fracture risk was overall bone strength by FEA.(29) The strongest predictors of fracture were UDR vBMD, cortical thickness, trabecular number, and axial rigidity (a measure of strength). The areas under ROC curves for these parameters and for aBMD at the UDR ranged from 0.55 to 0.66, similar to the our ROC analysis.(29)

In these women with central and peripheral fractures, we similarly found that trabecular stiffness based on FEA of HR-pQCT scans was associated with a history of fragility fracture. Women with fractures had significantly reduced Young's moduli and shear moduli at the radius and less pronounced reductions at the tibia. Since the tibia is a weight-bearing site, these results suggest that mechanical loading could be a key factor preventing trabecular bone loss. Furthermore, this difference may be related to the disparate microstructural changes that we observed at the two sites. At the radius, fracture subjects had significant reductions in trabecular number and increased trabecular network inhomogeneity. In contrast, at the tibia, trabecular number was the same in fracture subjects, whereas trabecular thickness, albeit a calculated rather than directly measured parameter, was lower. Guo and Kim demonstrated that trabecular loss is more detrimental to Young's modulus and strength of trabecular bone than trabecular thinning despite similarly decreased bone mass.(40) That men have preserved trabecular number but decreased thickness with age, whereas women have decreased trabecular number and increased separation has been used to explain the lower lifetime fracture risk in men.(23) Therefore, the reduction in trabecular number we observed at the radius is likely to be a strong determinant of the profound reduction in strength at that site.

The substantial reduction in radial stiffness observed in fracture subjects mirrors the markedly lower central trabecular density and suggests that the mechanism for fracture in our subjects could be related to preferential loss of inner trabeculae. The FEA technique we used evaluates only trabecular bone and does not include contributions from cortical bone. However, FEA of the cubic trabecular bone subvolume assesses important anisotropic material properties of the trabecular bone component. The accuracy of FEA of the HR-pQCT subvolume has been validated by micro–computed tomography (µCT)–based FEA, and excellent agreements were found for all six elastic moduli (*r*^2^ = 0.91–0.96).(26) Moreover, the estimated elastic moduli by subvolume FEA were significantly correlated with the stiffness of the whole-bone segment (*r*^2^ = 0.48–0.60) and trabecular bone segment (*r*^2^ = 0.53–0.70).(26) In future studies we will measure whole-bone FEA and assess mechanical competence of both cortical and trabecular bone to explore the contributions of both compartments to the differences in mechanical competence associated with fragility fractures.

In ROC analyses, we found that HR-pQCT did not perform better than DXA for fracture discrimination, and no DXA or HR-pQCT parameter demonstrated adequate discrimination for subjects with fractures. HR-pQCT may not have been superior to DXA because DXA measurements are less variable and are influenced by bone size. Further, it is conceivable that we have not yet found the ideal set of components to analyze with HR-pQCT. Further, although we observed substantial differences between fracture and nonfracture subjects for many of the structural and strength measures, there was overlap between the groups. As a result, it is not possible to extrapolate a threshold above which fracture susceptibility is high from these data. Our findings, similar to those of Melton and colleagues,(29) who reported *AUC*s for these parameters and for aBMD at the UDR ranging from 0.55 to 0.66, may reflect the many other factors that determine fracture risk and that are not measured by either of these techniques. These include mineralization and material properties of the bone, including BMD distribution, and collagen. Most important, fracture risk is related to propensity to fall, which cannot be assessed by any imaging technique.

Limitations of this work include the cross-sectional design, which precluded our assessment of the ability of HR-pQCT to predict fractures prospectively. Although we attempted to enroll subjects as close to the fracture event as possible, we were unable to evaluate many when fractures occurred. A potential limitation is that HR-pQCT assesses microstructure and volume at peripheral sites, and the fractures associated with the most significant morbidity and mortality are those which occur at central sites, namely, the hip and spine. In other work, we demonstrated correlations between FEA of vertebral bodies and of the radius and tibia,(35) as well as between aBMD of the LS and TH, with HR-pQCT of the radius and tibia.(15)

Unique strengths of this study are that it is the first to directly compare differences in radial and tibial measurements and to separately examine how central trabecular density varied between the two sites. We also performed FEA on a large group of subjects with multiple fracture types, thus extending FEA findings from previous in vivo studies, which focused on wrist fractures. We limited a number of potential confounders by excluding women with known secondary causes of bone loss, whereas other studies, using population-based cohorts, were unable to do so. In particular, by excluding women who had used bisphosphonates for more than 1 year, we avoided the possibility of artifact on the HR-pQCT scans from hypermineralization, which can occur after long-term bisphosphonate use(41) and may influence edge detection by HR-pQCT software.

In conclusion, we found that women with a diverse group of central and peripheral postmenopausal fragility fractures did not differ from those without fractures on the basis of DXA measurements, except at the ultradistal radius. In contrast, HR-pQCT at both the radius and the tibia revealed reduced vBMD and microarchitectural deterioration in women with a history of fracture. At the radius, the changes predominantly reflected trabecular dropout, with particularly substantial loss of inner trabecular density, reductions in trabecular number, and increased heterogeneity of the network. At the tibia, the most profound microarchitectural changes were in cortical thickness and trabecular thickness. FEA of a trabecular bone subvolume showed reduced stiffness, with the most marked reductions at the radius. While our findings suggest that HR-pQCT and FEA effectively discriminate fracture status in subjects with fragility fractures at multiple sites, by ROC analyses, we found that HR-pQCT was not superior to DXA. Longitudinal studies will be invaluable in comparing the abilities of DXA and HR-pQCT to predict future fractures in postmenopausal women and other at-risk populations. At present, HR-pQCT and FEA provide novel information regarding fracture mechanisms in postmenopausal women with osteoporosis.
